# Sidney Smith as a Country Doctor

**Published:** 1855-09

**Authors:** 


*Sidney Smith as a Country Doctor.*—"But I came up to speak to Annie Kay. Where is Annie Kay? Ring the bell for Annie Kay." Kay appeared. "Bring me my medicine book, Annie Kay. Kay is my apothecary's boy, and makes up my medicines." Kay appears with the book. "I am a great doctor; would you like to hear some of my medicines?" "Oh yes, Mr. Sidney." "Here is the gentle-joy, a pleasure to take it; the bull-dog, for more serious cases; Peter's puke; heart's delight, the comfort of all the old women in the village; rub-a-dub, a capital embrocation; dead-stop, settles the matter at once; up-with-it-then, needs no explanation; and so on. Now, Annie Kay, give Mrs. Spratt a bottle of rub-a-dub; and to Mr. Coles, a dose of dead-stop, and twenty drops of laudanum. This is the house to be ill in (turning to us;) indeed every body who comes is expected to take a little something; I consider it a delicate compliment when my guests have a slight illness here. We have contrivances for every thing. Have you seen my patent armour?" "No." "Annie Kay, bring my patent armour. Now, look here, if you have a stiff neck or swelled face, here is this sweet case of tin filled with hot water, and covered with flannel, to put round your neck, and you are well directly. Likewise, a patent tin shoulder, in case of rheumatism. Here you see a stomach-tin, the greatest comfort in life; and lastly, here is a tin slipper, to be filled with hot water, which you can sit with in the drawing-room, should you come in chilled, without wetting your feet. Come and see my apothecary's shop." We all went down stairs, and entered a room filled entirely on one side with medicines, and on the other with every description of groceries and household or agricultural necessaries; in the centre, a large chest, forming a table, and divided into compartments for soap, candles, salt, and sugar. "Here you see," said he, "every human want before you."—*From "A Memoir" by Lady Holland.*

